# Perfil e percurso profissional de egressos dos cursos de mestrado e doutorado da Fundação Oswaldo Cruz (2013-2020) 

**DOI:** 10.1590/0102-311XPT209222

**Published:** 2024-02-26

**Authors:** Suely Deslandes, Joviana Quintes Avanci Pina, Liana Wernersbach Pinto, Isabella Fernandes Delgado, Cosme Marcelo Furtado Passos da Silva

**Affiliations:** 1 Instituto Nacional de Saúde da Mulher, da Criança e do Adolescente Fernandes Figueira, Fundação Oswaldo Cruz, Rio de Janeiro, Brasil.; 2 Escola Nacional de Saúde Pública Sergio Arouca, Fundação Oswaldo Cruz, Rio de Janeiro, Brasil.; 3 Instituto Nacional de Controle de Qualidade em Saúde, Fundação Oswaldo Cruz, Rio de Janeiro, Brasil.

**Keywords:** Educação de Pós-graduação, Programa de Pós-graduação em Saúde, Avaliação Educacional, Graduate Education, Health Postgraduate Programs, Educational Measurement, Educación de Postgrado, Programas de Posgrado en Salud, Evaluación Educacional

## Abstract

O objetivo do estudo é analisar o perfil de egressos e identificar fatores associados ao desempenho positivo relativo à trajetória de formação e de inserção profissional entre aqueles que concluíram cursos presenciais de mestrado e doutorado acadêmico da Fundação Oswaldo Cruz (Brasil). Participaram do estudo 2.462 egressos (1.402 de mestrado e 1.060 de doutorado) que responderam um questionário digital, contendo questões sobre perfil do egresso e inserção profissional antes do ingresso no curso e após a conclusão. Foi criada uma medida sobre “desempenho positivo pós-curso”. Os resultados revelam que egressos de mestrado com impacto positivo do curso tendem a ser homens; ter especialização antes de ingressar no curso; ter emprego remunerado após o término do curso nas áreas de pesquisa, educação, assistência e gestão; e ser servidor público. No doutorado o seguinte perfil é evidenciado: egressos com emprego remunerado após o término do curso nas áreas de pesquisa ou educação; contratados por Regime Jurídico Único ou contrato temporário de pessoa jurídica; e com publicação científica ou patente. A avaliação/acompanhamento de egressos deve se instaurar como uma importante política pública educacional, incorporada no processo de autoavaliação institucional, o que possibilita rever rumos e prioridades na agenda educacional e pedagógica da instituição.

## Abstract (en)

This study aims to analyze the profile of graduates and identify factors associated with positive performance regarding training and professional insertion among those who have completed face-to-face master’s and doctoral courses at the Oswaldo Cruz Foundation (Brazil). A total of 2,462 graduates participated in the study (1,402 master’s and 1,060 doctoral graduates), who answered a digital questionnaire containing questions about individual characteristics and professional insertion before entering the course and after completion. A measure on “positive post-course performance” was created. The results show that Master’s graduates with a positive impact from the course tend to be men; to have a specialization degree before entering the course; to have paid employment after finishing the course in the areas of research, education, assistance, and management; and to be civil servants. In the Doctorate, the following profile is evident: graduates with paid employment after finishing the course in the areas of research or education; hired under a single legal regime or temporary contract as a legal entity; and with a scientific publication or patent. Evaluating/monitoring graduates must be established as an important public educational policy, incorporated into the institutional self-evaluation process, which makes it possible to review directions and priorities in the institution’s educational and pedagogical agenda.

## Resumen

El objetivo del estudio es analizar el perfil de egresados e identificar factores asociados al desempeño positivo relacionado a la trayectoria de formación e inserción profesional entre aquellos que concluyeron cursos presenciales de maestría y doctorado académico de la Fundación Oswaldo Cruz (Brasil). Participaron del estudio 2.462 egresados (1.402 de maestría y 1.060 de doctorado) que contestaron el cuestionario de manera digital, con preguntas sobre el perfil del egresado y la inserción profesional antes del ingreso al curso y tras su conclusión. Se creó una medida sobre “desempeño positivo tras el curso”. Los resultados muestran que los egresados de la maestría con un impacto positivo del curso tienden a ser hombres; tener especialización antes de ingresar al curso; tener trabajo remunerado tras concluir el curso en las áreas de investigación, educación, asistencia y gestión; y ser funcionario público. Se evidencia el siguiente perfil en el doctorado: egresados con trabajo remunerado tras el término del curso en las áreas de investigación o educación; contratados bajo un régimen jurídico único o un contrato temporal de persona jurídica; y con publicación científica o patente. La evaluación/seguimiento de egresados se debe establecer como una importante política pública educativa, incorporada en el proceso de autoevaluación institucional, lo que permite revisar direcciones y prioridades en la agenda educativa y pedagógica de la institución.

## Introdução

No contexto dos debates quanto ao papel, a finalidade e a qualidade da formação no Ensino Superior e na pós-graduação brasileira, estudos de acompanhamento de egressos se colocam como estratégicos na reflexão sobre a qualificação do conhecimento e a efetividade da proposta de ensino, pesquisa e ações sociais e culturais em instituições de Ensino Superior do país ^1^.

Desde 2017, a Coordenação de Aperfeiçoamento de Pessoal de Nível Superior (CAPES), em um intenso movimento de reconfiguração de seu modelo avaliativo, reconhece a necessidade de acompanhamento de egressos da pós-graduação *stricto sensu*, considerando a trajetória acadêmica e profissional como indicativo da qualidade na formação de doutores e mestres do país. No mesmo período, em 2018, o documento final da Comissão Nacional de Acompanhamento do Programa Nacional de Pós-graduação ressalta a importância do acompanhamento dos egressos, considerando as peculiaridades de cada área de conhecimento, e destaca a titulação como consequência do processo formativo e a forte relação entre esse acompanhamento e o aperfeiçoamento de políticas institucionais ^2^.

A percepção de egressos tem sido considerada uma fonte relevante de informação para a avaliação de programas de pós-graduação *stricto sensu* e suas estratégias de formação, assim como a análise de sua inserção profissional, posições ocupadas no mercado de trabalho e a maior qualificação das atividades do trabalho ^1^^,^^3^^,^^4^^,^^5^. A estreita relação do processo formativo com o mundo do trabalho é marcante nesses programas, nos quais há uma expectativa social e institucional de prover uma formação que possibilite aprofundamento e domínio de conhecimentos na área de interesse, bem como preparar o(a) aluno(a), mestre(a) ou doutor(a) para exercício profissional altamente qualificado ^1^^,^^6^.

No Brasil, é evidente a escassez de estudos sistematizados sobre o acompanhamento de programas e, mais ainda, de egressos de cursos de pós-graduação ^7^, o que poderia nortear a reflexão sobre experiências bem-sucedidas, incrementar estratégias de progresso científico, subsidiar o fortalecimento da pós-graduação brasileira e favorecer o entendimento acerca do papel social de instituições de Ensino Superior ^8^. Nesse sentido, a avaliação é a ferramenta principal para a organização e implementação das reformas educacionais, fornecendo subsídios para mudanças nos currículos, nas metodologias de ensino, nos conceitos e práticas de formação, na gestão, nas configurações do sistema educativo, nas políticas e prioridades da pesquisa, e nas noções de pertinência e responsabilidade social ^9^^,^^10^.

A Fundação Oswaldo Cruz (Fiocruz) é um órgão do Ministério da Saúde e uma instituição de ciência e tecnologia dedicada à pesquisa, ao ensino e ao desenvolvimento tecnológico no campo da saúde. Tem como missão: a formação de quadros de elevada competência; redução das desigualdades na formação qualificada para o Sistema Único de Saúde (SUS) e o Sistema de Ciência, Tecnologia e Inovação (CT&I); o fortalecimento da internacionalização da educação; o compromisso com o acesso aberto ao conhecimento e “ciência aberta”; e o fortalecimento da comunicação e divulgação científica ^11^. Todas as suas unidades técnico-científicas desenvolvem programas de pós-graduação *stricto sensu*, inseridos em dez áreas de avaliação da CAPES. Em 1976, o Programa em Biologia Parasitária (Instituto Oswaldo Cruz) foi o primeiro da instituição a ser credenciado pela CAPES.

Considerando registros mais recentes, desde 2004, a Fiocruz formou 12.856 alunos no nível do *stricto sensu*, sendo 3.769 de doutorado acadêmico, 6.682 de mestrado acadêmico e 2.405 de mestrado profissional. A partir de 2019, iniciou-se a implantação do sistema de acompanhamento dos egressos (*stricto sensu*, especializações e residências) como componente do sistema de autoavaliação e de uma política de egressos na instituição. A análise de egressos é estratégica não só por sua magnitude, mas também pelo histórico papel de destaque que a Fiocruz tem na pós-graduação brasileira e na formação no campo da saúde.

Assim, este estudo tem como objetivo analisar o perfil de egressos e identificar fatores associados ao desempenho positivo relativo à trajetória de formação e de inserção profissional entre aqueles que concluíram cursos presenciais de mestrado e doutorado acadêmico da Fiocruz.

## Método

### População

O estudo parte do universo de 3.775 egressos (2.235 de mestrado e 1.540 de doutorado) de 25 cursos presenciais de pós-graduação *stricto sensu* da Fiocruz, concluídos entre janeiro de 2013 e julho de 2020 e distribuídos em 12 unidades da instituição no país ^12^. Fizeram parte deste estudo 2.462 egressos (1.402 de mestrado e 1.060 de doutorado), correspondendo a cerca de 65% do total de convidados elegíveis a participar (Tabela 1). As listas dos egressos de cada curso/unidade foram obtidas por meio da plataforma Sistema de Gestão Acadêmica (Siga/Fiocruz; https://www.siga.fiocruz.br/) e atualizadas com a secretaria acadêmica e coordenações de cada programa.

**Tabela 1:** Número de egressos participantes dos cursos de mestrado e doutorado acadêmicos por unidades da Fundação Oswaldo Cruz (Fiocruz) (N = 2.462).

Unidades da Fiocruz	Mestrado		Doutorado	
	n	%	n	%
Casa de Oswaldo Cruz (COC)	85	6,1	31	2,9
Escola Nacional de Saúde Pública Sergio Arouca (ENSP)	348	24,8	287	27,1
Fiocruz Amazônia - Instituto Leônidas e Maria Deane (ILMD)	47	3,4	0	0,0
Fiocruz Bahia - Instituto Gonçalo Moniz (IGM)	60	4,3	38	3,6
Fiocruz Minas - Instituto René Rachou (IRR)	83	5,9	97	9,2
Fiocruz Paraná - Instituto Carlos Chagas (ICC)	25	1,8	16	1,5
Fiocruz Pernambuco - Instituto Aggeu Magalhães (IAM)	112	8,0	86	8,1
Instituto de Comunicação e Informação Científica e Tecnológica em Saúde (ICICT)	62	4,4	37	3,5
Instituto Nacional de Controle de Qualidade em Saúde (INCQS)	62	4,4	36	3,4
Instituto Nacional de Infectologia Evandro Chagas (INI)	62	4,4	71	6,7
Instituto Nacional de Saúde da Mulher, da Criança e do Adolescente Fernandes Figueira (IFF)	77	5,5	45	4,2
Instituto Oswaldo Cruz (IOC)	379	27,0	316	29,8

### Medidas utilizadas

Foi desenvolvido um questionário digital por meio do LimeSurvey (https://www.limesurvey.org/pt-br), que é um software de código aberto utilizado para a elaboração e aplicação de questionários *online*. Por e-mail, cada egresso recebeu um *link* de acesso ao seu questionário com uma chave de acesso individual. Uma ampla campanha de divulgação da pesquisa foi realizada nos *sites* das unidades, no campus virtual da Fiocruz, nas redes sociais (Instagram e Facebook), em listas de WhatsApp e por publicação na revista *Radis* da Escola Nacional de Saúde Pública Sergio Arouca (ENSP)/Fiocruz ^12^.

Durante um período de três meses, e-mails de convite eram enviados, semanalmente, para aqueles que ainda não tinham preenchido o questionário. Esse acompanhamento do número de respondentes possibilitou que os gestores de ensino e orientadores de cada unidade se mobilizassem para o contato com os ex-alunos de forma a os sensibilizarem a participar do estudo. Foi criado um e-mail específico endereçado aos egressos e interessados na pesquisa para facilitar a comunicação e esclarecer dúvidas.

As questões abordadas no questionário eram relativas à avaliação de egressos e contou com discussões com especialistas em gestão e avaliação de ensino, chegando a uma versão preliminar. A versão final do questionário foi testada e aplicada a uma amostra de cerca de 10% de egressos de uma unidade eleita por conveniência. Nessa ocasião, verificou-se boa compreensão das questões e tempo de preenchimento satisfatório, que variou entre 10 e 15 minutos ^12^.

O questionário foi composto por 42 questões de múltipla escolha, distribuídos em seis blocos temáticos: identificação do egresso e do curso; atividade profissional antes de ingressar no curso; atividade profissional e expectativas logo após terminar o curso; condição empregatícia atual; efeitos da formação; e avaliação da trajetória formativa ^12^. O questionário foi publicizado e disponibilizado para acesso livre pelo repositório institucional da Fiocruz: Arca (https://www.arca.fiocruz.br/handle/icict/36744).

As seguintes variáveis foram investigadas: (1) perfil do egresso: sexo; idade; cor de pele autodeclarada; deficiência; inserção por cota; país e estado em que reside; área de formação na graduação ^13^; área do curso *stricto sensu* realizado segundo classificação pelos Colégios da CAPES; ter outra formação acadêmica no momento de ingresso e expectativas ao concluir o curso; e (2) inserção profissional antes do ingresso no curso e após a conclusão: se estava trabalhando; área de atuação; local de trabalho; regime de contratação; aumento de salário; mudança de atividade profissional em decorrência do curso e relação da atividade profissional com o curso.

Uma medida sobre o desempenho positivo pós-curso foi criada a partir da variável efeito do título na vida profissional, em que se considerou a resposta positiva a qualquer dos seguintes itens: “o curso qualificou para um melhor desempenho das atividades que já exercia ou para atividades diferentes” ou “o curso aumentou o prestígio e reconhecimento do trabalho diante de colegas e chefia”.

### Análise dos dados

Inicialmente, foi realizada descrição da frequência absoluta e relativa das variáveis de perfil e inserção profissional antes de ingresso no curso e após o seu término. Posteriormente, foi avaliada a relação das variáveis relativas à inserção profissional e quantidade de emprego após o término dos cursos de mestrado e doutorado, segundo sexo, cor da pele e Colégio da CAPES (Ciências da Vida, Humanidades e Ciências Exatas, Tecnológicas e Multidisciplinar), comparando as proporções por meio do teste de qui-quadrado, ao nível de 5% de significância.

Para a verificação dos fatores associados ao desempenho positivo pós-curso de mestrado e doutorado acadêmicos, foram ajustados modelos de regressão logística, sendo empregado o método *backward* manual para a inclusão de variáveis. No modelo múltiplo, foram inseridas as variáveis explicativas com p < 0,25 nos modelos simples pelo teste da razão de verossimilhança. Na avaliação da qualidade do ajuste dos modelos, utilizou-se o teste de Hosmer & Lemeshow ^14^.

Para identificar as variáveis associadas ao desempenho positivo pós-curso, a amostra analisada foi composta por 810 egressos de mestrado e 753 de doutorado, que concluíram o curso entre 2013 e 2018 e que se autodenominam brancos, pardos e pretos. Essa opção se deu pelo número muito reduzido de participantes que se autodenominam de cor de pele amarela ou indígena, o que dificultaria a análise estatística, e pela dificuldade em agregá-los aos demais perfis étnico-raciais. Foram excluídos, ainda, da etapa de modelagem, os egressos de 2019 e 2020 por não serem solicitados a responder questões sobre a inserção profissional, por terem acabado de sair do curso e, portanto, haver pouco tempo de impacto do curso em sua trajetória profissional. Foram consideradas as seguintes variáveis independentes: sexo (homem; mulher); idade (≤ 30 anos; 31-50 anos; > 50 anos); cor da pele (branca; preta ou parda); tempo de conclusão do curso (em anos); se tem alguma formação de pós-graduação (qualificação profissional/aperfeiçoamento ou especialização ou residência ou mestrado profissional ou mestrado acadêmico ou doutorado; não); regime de contração de trabalho após a conclusão do curso (*Consolidação das Leis de Trabalho* [CLT] ou contrato temporário como pessoa física ou cooperativa ou cargo comissionado ou autônomo ou bolsista; Regime Jurídico Único ou contrato temporário como pessoa jurídica ou empresa própria); quantidade de empregos (nenhum; um ou mais); área de atuação (assistência; gestão; educação; pesquisa - sim; não; não trabalha); local da atividade de trabalho (público; privado; autônomo ou terceiro setor; não trabalhava); mudança de atividade profissional (sim; não; não trabalhava); relação da atividade profissional com o curso (muito relacionada; razoavelmente relacionada; pouco relacionada ou não tem relação; não trabalha); e aumento salarial após a conclusão do curso (sim; não; não trabalhava). Foram calculadas as razões de chance e os intervalos de 95% de confiança (IC95%). Toda a análise foi realizada no software IBM SPSS Statistics, versão 24 (https://www.ibm.com/).

### Cuidados éticos - confidencialidade

Este estudo foi concebido como um levantamento em um nível de gestão. Os dados utilizados são de um banco de dados público, dispensando, assim, submissão ao Comitê de Ética em Pesquisa. Todavia, cabe ressaltar que todos os cuidados éticos previstos sobre confidencialidade e autonomia de participação foram garantidos, conforme as resoluções vigentes do Conselho Nacional de Saúde.

## Resultados

A Tabela 2 mostra o perfil dos egressos dos cursos de mestrado e doutorado. Observa-se que, independentemente do curso, a maior parte dos participantes são do sexo feminino (74,3% no mestrado e 73,1% no doutorado), têm até 30 anos de idade (67% no mestrado e 45,9% no doutorado), se autodeclaram de cor de pele branca (59,9% no mestrado e 66,4% no doutorado), informam não ter deficiência (cerca de 99% em cada curso) e residem no Brasil (praticamente 97% em cada curso), predominantemente no Estado do Rio de Janeiro. Uma parcela pequena de egressos do mestrado (0,7%) ingressou no curso por cota racial. A área de ciências da saúde e bem-estar é a formação na graduação da maioria dos participantes (48,2% no mestrado e 49,2% no doutorado), seguida da área de ciências naturais, matemática e estatística (cerca de 30% em cada curso). Cerca de 45% dos egressos de mestrado e doutorado já tinham curso de especialização antes de ingressar nos cursos *stricto sensu*. Contudo, no mestrado, é expressivo o número de participantes que não tinham formação acadêmica anterior. Continuar a estudar (44,9%) é a maior expectativa dos egressos do mestrado ao terminarem o curso, ao passo que atuar em grupo de pesquisa (52,5%) e atuar no serviço público de forma mais qualificada (40,3%) são as maiores aspirações entre os egressos de doutorado.

**Tabela 2:** Perfil dos egressos de cursos de mestrado (N = 1.402) e doutorado (N = 1.060) acadêmicos da Fundação Oswaldo Cruz (Fiocruz).

Variáveis	Mestrado		Doutorado	
	n	%	n	%
Sexo				
Feminino	1.041	74,3	775	73,1
Masculino	358	25,5	284	26,8
Outros	3	0,2	1	0,1
Idade (anos)				
≤ 30	940	67,0	486	45,9
31-40	336	24,0	355	33,5
41-50	98	7,0	154	14,5
≥ 51	28	2,0	65	6,1
Cor da pele				
Branca	840	59,9	704	66,4
Parda	388	27,7	270	25,5
Preta	140	10,0	70	6,6
Amarela	25	1,8	11	1,0
Indígena	9	0,6	5	0,5
Possui alguma deficiência				
Sim	14	1,0	14	1,3
Não	1.388	99,0	1.046	98,7
Inserção por cota				
Racial	10	0,7	-	-
Deficiência	-	-	1	0,1
Não	1.392	99,3	1.059	99,9
País onde morava				
Brasil	1.359	96,9	1.031	97,2
Moçambique	15	1,1	4	0,4
Peru	7	0,5	1	0,1
Colômbia	6	0,4	8	0,8
Argentina	6	0,4	-	-
Cuba	-	-	3	0,3
Uruguai	-	-	2	0,2
Paraguai	-	-	2	0,2
Bolívia	-	-	2	0,2
Demais países	9	0,7	7	0,6
Estado que morava antes do curso				
Rio de Janeiro	879	62,7	620	58,5
Minas Gerais	118	8,4	122	11,5
Pernambuco	98	7,0	84	7,9
Bahia	67	4,8	47	4,4
Amazonas	48	3,4	14	1,3
Espírito Santo	3	0,2	20	1,9
Paraná	23	1,6	17	1,6
Acre	1	0,1	16	1,5
Demais estados	122	8,7	91	8,6
Não se aplica	43	3,1	29	2,8
Área de formação na graduação				
Ciências da saúde e bem-estar	676	48,2	521	49,2
Ciências naturais, matemática e estatística	409	29,1	339	32,0
Ciências sociais, jornalismo e informação	160	11,4	83	7,8
Artes e humanidades	47	3,4	28	2,6
Agricultura, silvicultura, pesca e veterinária	47	3,4	55	5,2
Demais áreas	63	4,5	34	3,2
Outra formação acadêmica no ingresso ao curso *				
Não tem	538	38,4	40	3,8
Qualificação profissional ou aperfeiçoamento	205	14,6	219	20,7
Especialização	636	45,4	464	43,8
Residência	254	18,1	122	11,5
Mestrado profissional	5	0,4	65	6,1
Mestrado acadêmico	42	3,0	926	87,4
Doutorado acadêmico	56	4,0	62	5,8
Expectativa ao término do curso *				
Continuar a estudar	630	44,9	193	18,2
Atuar como docente na graduação e/ou programa de pós-graduação	547	39,0	636	60,0
Atuar em grupo de pesquisa	451	32,2	556	52,5
Atuar no setor público de forma mais qualificada	432	30,8	427	40,3
Continuar a estudar, após organizar melhor a vida profissional	431	30,7	150	14,2
Ingressar no setor público	345	24,6	360	34,0
Obter melhores rendimentos	335	23,9	388	36,6
Atuar no setor privado de forma mais qualificada	152	10,8	92	8,7
Ingressar no setor privado	115	8,2	77	7,3
Atuar no setor privado de forma mais competitiva	74	5,3	46	4,3
Ser promovido	62	4,4	79	7,5
Não tinha expectativas	16	1,1	12	1,1

* Questões de resposta múltipla.

A Tabela 3 apresenta as características da inserção profissional dos egressos antes e após a conclusão dos cursos. Em comparação ao número de egressos que trabalhavam antes de iniciar o curso, fica muito evidente a importância da formação em cursos *stricto sensu* na inserção no mercado de trabalho. Observa-se incremento de cerca de 25% de egressos que passaram a trabalhar após o término do mestrado e aproximadamente 20% do doutorado. Cerca de 26% dos egressos de mestrado não estavam trabalhando após o término do curso e um número bem pequeno (9,8%) daqueles oriundos do doutorado refere estar nessa condição. As áreas de pesquisa e educação se destacam como campos de atuação entre os egressos do mestrado e, sobretudo, do doutorado, seja antes ou após a conclusão dos cursos. A atuação na pesquisa denota ser a área que teve mais migração de alunos após o término do curso, especialmente entre os de doutorado.

**Tabela 3:** Inserção profissional dos egressos dos cursos de mestrado e doutorado acadêmicos da Fundação Oswaldo Cruz (Fiocruz).

Variáveis	Mestrado (%)		Doutorado (%)	
	Antes do curso (n = 1.402)	Depois do curso * (n = 1.119)	Antes do curso (n = 1.060)	Depois do curso * (n = 849)
Estava trabalhando				
Sim	59,3	74,1	69,2	90,2
Não	40,7	25,9	30,8	9,8
Área de atuação **				
Pesquisa	11,6	23,1	22,7	54,1
Educação	13,1	23,0	32,4	49,6
Assistência	21,8	21,9	19,5	17,9
Comunicação	3,5	2,8	1,6	1,1
Gestão	9,1	12,2	11,0	10,5
Produção de insumos	1,2	2,3	1,2	2,1
Produção de bens e serviços	0,9	2,2	1,1	1,4
Ativismo social	0,6	0,5	0,8	0,7
Não trabalhavam	40,7	20,7	30,8	9,8
Local de trabalho				
Instituição pública	39,5	54,0	52,5	74,2
Instituição privada	14,2	14,7	13,7	13,2
Autônomo ou terceiro setor/sociedade civil/ONG/OS	4,9	5,4	2,9	2,8
Outros	0,7	-	0,1	-
Não trabalhavam	40,7	25,9	30,8	9,8
Regime de contratação				
CLT	17,1	19,8	15,1	15,9
Regime Jurídico Único	15,5	21,6	30,4	43,6
Contrato temporário pessoa física	6,0	4,9	6,4	6,4
Bolsista	7,7	11,7	6,4	12,0
Cargo comissionado	1,6	1,4	1,0	0,7
Autônomo	3,4	4,1	2,5	2,2
Empresa própria	0,7	1,5	1,2	0,2
Contrato temporário pessoa jurídica	0,1	0,7	0,5	0,4
Cooperativa	0,4	0,2	0,3	-
Outros	6,8	8,2	5,4	8,8
Não trabalhavam	40,7	25,9	30,8	9,8
Aumento salarial				
Sim	-	36,4	-	68,1
Não	-	32,9	-	17,2
Não sabe dizer	-	4,8	-	4,9
Não trabalhavam	-	25,9		9,8
Mudança de atividade profissional em decorrência do curso				
Sim	-	18,0	-	27,2
Não	-	16,9	-	12,0
Não sabe dizer	-	2,8	-	4,5
Não mudou em decorrência do curso	-	36,4	-	46,5
Não trabalhavam	-	25,9		9,8
Relação da atividade profissional com o curso				
Muito relacionada	-	39,9	-	65,8
Razoavelmente relacionada	-	20,3	-	16,1
Pouco relacionada	-	8,0	-	5,8
Não tem relação	-	5,9	-	2,5
Não trabalha	-	25,9	-	9,8

CLT: *Consolidação das Leis do Trabalho*; ONG: organizações não governamentais; OS: organizações sociais.

* Os alunos que finalizaram o curso no ano de preenchimento não preencheram o bloco 5;

** Questão de resposta múltipla.

A instituição pública é o local de trabalho que prevalece entre egressos dos cursos de mestrado ou doutorado, antes ou depois de sua conclusão. Contudo, supõe-se uma mobilidade de ascensão laboral do egresso dentro do serviço público em decorrência da qualificação profissional pós-curso no mesmo nível de gestão ou, especialmente, em nível distinto (migração do municipal-estadual ou estadual-federal, por exemplo). O vínculo empregatício de Regime Jurídico Único e CLT se sobressaem nos dois cursos. Contudo, ao observar os achados de antes e depois da conclusão do curso, o Regime Jurídico Único e a bolsa são os que apresentam maior aumento de egressos após o término dos cursos, na comparação com o vínculo de entrada. É bem expressivo o aumento de salário após a conclusão dos cursos: 36,4% assim afirmam após o término do mestrado e 68,11% após o doutorado. A mudança de atividade profissional após a conclusão do curso foi relatada por 27,2% dos egressos de doutorado e 18% do mestrado. A constatação de boa parte dos egressos, que relatam muita relação do curso com sua atividade profissional, revela a forte interdependência da trajetória acadêmica com a profissional.

Na análise da inserção profissional após a conclusão do curso segundo o sexo, destaca-se que muito mais mulheres do curso de mestrado estavam fora do mercado de trabalho após o término do curso (73% contra 37,7% dos homens) e mais homens tinham dois empregos (27,8% contra 19,1% das mulheres). Não se observa diferença estatística significativa da inserção no mercado de trabalho e quantidade de emprego segundo cor da pele. Não ter emprego e atuar em mais de dois foram bem mais presentes entre os egressos de cursos de mestrado do Colégio de Ciências da Vida (dados não apresentados).

A Tabela 4 mostra as razões de chance estimadas pelos modelos de regressão logística simples e múltipla buscando identificar as variáveis associadas ao impacto positivo do curso de mestrado e de doutorado.

**Tabela 4:** Modelo logístico explicativo do impacto positivo dos cursos de mestrado (n = 810) e doutorado (n = 753) acadêmicos entre egressos da Fundação Oswaldo Cruz (Fiocruz).

Variável	OR bruta	IC95%	OR ajustada	IC95%
Mestrado acadêmico				
Sexo				
Masculino	2,004	1,332-3,017	1,885	1,229-2,892
Feminino	-	-	-	-
Formação anterior (especialização)				
Sim	1,472	1,056-2,051	1,642	1,142-2,361
Não	-	-	-	-
Emprego remunerado na área de pesquisa				
Sim	2,452	1,625-3,698	3,325	2,066-5,350
Não	-	-	--	--
Emprego remunerado na área de educação				
Sim	1,675	1,142-2,455	2,134	1,407-3,237
Não	-	-	-	-
Emprego remunerado na área de assistência				
Sim	1,265	0,871-1,836	1,888	1,242-2,870
Não	-	-	-	-
Emprego remunerado na área de gestão				
Sim	2,176	1,270-3,729	2,778	1,544-4,997
Não	-	-	-	-
Local onde exerce				
Público	2,443	1,725-3,459	1,851	1,266-2,706
Privado	-	-	-	-
Doutorado acadêmico				
Emprego remunerado na área de pesquisa				
Sim	3,385	2,122-5,400	3,795	2,237-6,188
Não	-	-	-	-
Emprego remunerado na área de educação				
Sim	2,164	1,374-3,409	2,095	1,298-3,382
Não	-	-	-	-
Regime de contratação				
Regime Jurídico Único ou contrato temporário de pessoa jurídica	2,237	1,423-3,518	2,382	1,471-3,857
CLT ou contrato temporário pessoa física	-	-	-	-
Produção				
Publicou artigo, livro, capítulo ou patente	2,320	1,348-3,995	2,134	1,196-3,807
Não produziu esses materiais	-	-	-	-

CLT: *Consolidação das Leis do Trabalho*; IC95%: intervalo de 95% de confiança: OR: *odds ratio*.

O modelo final mostra o seguinte perfil de egressos com impacto positivo do curso de mestrado concluído: homens têm cerca de 88% mais chance de ter impacto positivo do curso do que as mulheres; os que têm curso de especialização antes de ingressar no curso têm 64,2% mais probabilidade do que aqueles que não têm formação anterior; aqueles que têm emprego remunerado na área de pesquisa têm 3,3 vezes a possibilidade dos que não têm essa atuação; os que têm inserção profissional nas áreas de educação e assistência têm, respectivamente, 113,4% e praticamente 88,8% mais chance do que aqueles que não têm essas inserções profissionais; aqueles que estão na área de gestão têm 2,8 vezes a oportunidade daqueles que não desenvolvem essa atuação; e os inseridos em trabalho de serviço público tem quase o dobro da chance de impacto positivo do curso em comparação àqueles em trabalho privado.

O impacto positivo do curso de doutorado se evidencia pelo seguinte perfil: egressos com emprego remunerado na área de pesquisa têm 3,8 vezes a chance daqueles sem essa atuação profissional; também aqueles inseridos na área de educação têm 109,5% mais oportunidades; aqueles com contratação de Regime Jurídico Único ou contrato temporário de pessoa jurídica têm 2,4 vezes a chance daqueles com regime CLT ou contrato temporário pessoa física; e os egressos que publicaram de artigo científico, livro, capítulo ou patente têm 113,4% mais probabilidade de impacto positivo do curso do que aqueles que não tiveram nenhuma publicação (Tabela 4).

## Discussão

O estudo revela que a formação oferecida pela instituição corresponde positivamente aos eixos que orientam a avaliação dos programas de pós-graduação *stricto sensu* do país, no sentido de apresentarem uma formação comprometida com as instituições públicas e com claro impacto social ^5^. A partir da perspectiva do ex-aluno(a), os achados mostram o importante efeito pós-curso na inserção laboral, especialmente nos nichos de ensino e pesquisa e, secundariamente, nos níveis de renda dos egressos, evidenciado pelo menor número de desempregados depois da formação, maior inserção nas áreas de educação e pesquisa, maior atuação no serviço público e o destaque para vínculo empregatício de Regime Jurídico Único. Essa inserção significativa do egresso com qualificações para o mercado de trabalho indica o cumprimento da “missão” de uma formação de excelência, que é aliada à reputação e ao prestígio institucional. Estudos de acompanhamento e avaliação de egressos, em especial aqueles oriundos de cursos de graduação e mestrado profissional, também têm demonstrado mais resultados positivos em quesitos de desempenho e crescimento pessoal e profissional do que na remuneração e condições de trabalho ^3^^,^^15^^,^^16^. Além disso, é constatado o incremento na autoestima, nas habilidades de comunicação, na relevância social do trabalho, na oportunidade de novas aprendizagens e no exercício de criatividade ^3^^,^^16^^,^^17^. Cabe destacar que, embora os resultados apresentados sejam muito positivos, a formação acadêmica de qualidade vai além do preparo profissional para a atuação no mercado de trabalho. Envolve a formação como cidadão político e ético, ponto ainda mais decisivo em cursos da área da saúde ^18^^,^^19^. Além disso, o resultado da formação acadêmica exige a corresponsabilização do aluno(a) no processo formativo, no qual é autônomo, agente e autor do conhecimento.

A preponderância de egressos que estão inseridos no serviço público, bem como na pesquisa e no ensino, coincide com outros estudos ^15^^,^^20^ e indica que os cursos respondem positivamente à missão de formação qualificada para atuação no setor público e contribuem para a provisão de pesquisadores para o exercício da atividade acadêmica e científica. Em contrapartida, o crescimento do número de egressos com vínculo de bolsa após o curso mostra a fragilidade dos vínculos empregatícios aliada à incipiência de contratações via concurso público, num contexto de importante redução de investimento federal na ciência e tecnologia a partir de 2013. Entre 2013 e 2020, houve uma diminuição orçamentária de cerca de 37%, chegando, em 2020, a um nível inferior ao observado em 2009 ^21^. Situação semelhante ocorre com o Ministério da Educação (MEC), no qual se insere a principal agência responsável pela formação de cientistas do país: a CAPES. Neves ^22^ faz um levantamento a partir do Sistema Integrado de Administração Financeira do Governo Federal e mostra que o orçamento da CAPES sofreu redução, no período de 2015 a 2020, de mais de 50%, ou seja, de R$ 7,43 bilhões passou para R$ 3,54 bilhões. Esse difícil contexto tem impacto direto na formação e na atuação de pesquisadores brasileiros, no Brasil e no exterior.

Os achados também comprovam a marcante desigualdade de gênero no que se refere ao impacto positivo do curso entre os egressos de mestrado, com uma chance bem superior de os homens atingirem melhores resultados profissionais. Mesmo depois das mulheres conseguirem vencer as barreiras de acesso à carreira acadêmica, elas não avançam profissionalmente na mesma proporção e velocidade que os homens, o que pode ser explicado pela histórica sobrecarga do trabalho doméstico e cuidado com a prole e a família, mas, principalmente, por fatores de misoginia e discriminação de gênero ^23^^,^^24^. Nos espaços de trabalho remunerado, as trabalhadoras ainda estão em situação de desvantagem e desigualdade ^25^. A necessidade de implantação de uma política afirmativa de gênero desafia os serviços e as instituições de ensino a pensar em ações numa perspectiva interseccional, voltada às mulheres e, especialmente, às mulheres negras/pardas e indígenas.

Em uma perspectiva de desigualdade étnico-racial, os resultados revelam o escasso acesso de indígenas em cursos de pós-graduação *stricto sensu*, embora a Fiocruz tenha ofertas em estados com comunidades indígenas, o que poderia favorecer o ingresso desse público. Lima ^26^ explica que a pauta da luta pela diversidade indígena não é apenas uma luta pela inclusão, mas pelo debate de problemas epistemológicos e conceituais de natureza sociocultural, na qual os caminhos para a equidade entre indígenas e não indígenas são bastante distintos, e a instituição de ensino precisa adquirir conhecimentos sobre esses povos a fim de facilitar seu acesso, sua permanência e a finalização do curso. Similar escassez de acesso é observado para pessoas com deficiência, já que são necessárias adaptações de estruturas físicas e estratégias pedagógicas.

Desde 2017, a Fiocruz tem adotado ações afirmativas, com foco na diminuição das desigualdades étnico-raciais e na inclusão das pessoas com deficiências. As *Portarias nº 1.433/2017*^27^, *nº 6.162/2019*^28^ e *nº 491/2021*^29^ regulamentam que todos os cursos da Fiocruz devem destinar um número mínimo de 7% das vagas para candidatos que se declararem pessoas com deficiência, 20% aos que se autodeclararem negros (pretos e pardos) e 3% aos que se autodeclararem indígenas. Além disso, o Comitê Pró-equidade de Gênero e Raça e o Comitê de Acessibilidade e Inclusão da Pessoa com Deficiência fazem parte da agenda institucional para o fortalecimento dos temas étnico-raciais e de gênero na Fiocruz, colaborando para atualização e reorientação de suas políticas, bem como de suas ações nas relações de trabalho, no atendimento ao público e na produção e popularização do conhecimento. Todavia, essas ações são recentes e os resultados mostram a necessidade de investimento na área.

Por fim, reiteramos que a avaliação/acompanhamento de egressos deve se instaurar como uma importante política institucional em todas as instituições de Ensino Superior, incorporando os ex-alunos(as) como protagonistas nos processos de autoavaliação institucional, o que possibilita identificar acertos/potencialidades, insuficiências/dificuldades e rever rumos e prioridades na agenda educacional e pedagógica da instituição ^30^^,^^31^.

A principal limitação do estudo é a não garantia de generalização dos achados, uma vez que não é uma amostra probabilística, embora se tenha alcançado um número satisfatório de respondentes. Em contrapartida, o estudo contribui imensamente para a análise do desempenho profissional a partir de um conjunto diverso de egressos, possibilitando um conhecimento mais aproximado do impacto do curso a médio e longo prazo.
